# Managing and measuring performance of health prevention services: a simulation-based approach

**DOI:** 10.1108/JHOM-10-2024-0422

**Published:** 2025-05-22

**Authors:** Guido Noto, Francesca De Domenico, Sandra C. Buttigieg, Gustavo Barresi

**Affiliations:** Department of Economics, University of Messina, Messina, Italy; Department of Health Systems Management and Leadership, Faculty of Health Sciences, University of Malta, Msida, Malta; College of Social Sciences, University of Birmingham, Birmingham, UK

**Keywords:** Prevention, Performance management, Simulation, System dynamics, Healthcare

## Abstract

**Purpose:**

This study focuses on the application of performance management (PM) in health prevention services. Unlike other healthcare services that focus on individual health results, prevention activities aim at community-wide benefits, often related to the avoidance of negative health outcomes. This, coupled with delayed effects of prevention activities, external influences on results and multiple stakeholders, poses challenges for the management, measurement and accountability of the results achieved by healthcare organisations and systems. To address these challenges, the research proposes the adoption of simulation techniques, specifically system dynamics (SD), to enhance PM in the prevention sector.

**Design/methodology/approach:**

SD is a methodological approach developed for modelling and simulating complex systems and experimenting with the models to design strategies and policies. It provides a systemic perspective and a set of conceptual tools that enable one to frame the structure and behaviour of complex, nonlinear, multi-loop feedback systems through an illustrative case focused on the management of primary and secondary prevention of chronic care conditions within a Beveridge healthcare system.

**Findings:**

By employing SD, the study aims to provide decision-makers with the capability to understand the link between immediate outputs and long-term outcomes, facilitating the evaluation of alternative policy options and scenarios that are otherwise untestable due to the long latency of diseases, delayed impact of preventive actions and systemic fragmentation.

**Originality/value:**

Through the development of an SD model, this research contributes to the field by offering a novel approach to overcoming the measurement and accountability obstacles in prevention as part of healthcare PM.

## Introduction

Performance management (PM) in the healthcare sector has been widely adopted and studied since New Public Management reforms implementation (Nuti *et al.*, 2018; Vainieri *et al.*, 2020; Anders, 2024). According to an instrumental view, performance has been framed and measured in terms of resources, outputs and outcomes (Van Peursem *et al.*, 1995; Bianchi, 2010).

Although some healthcare settings, such as the acute hospital one, successfully managed to implement PM practices and have largely benefited from their adoption (Aidemark, 2001; Lega and Vendramini, 2008; Elg *et al.*, 2013; Anders, 2024), prevention services have struggled in following suit. This generated a significant gap in the application of PM frameworks to public health and prevention services (Landrum and Baker, 2004; Cinquini *et al.*, 2014; Schwartz and Deber, 2016). The reasons are multiple and related to the intrinsic complexity – or *wickedness* – of prevention activities, both primary and secondary (Schwartz and Deber, 2016; Noto *et al.*, 2023).

One of the key obstacles in managing performance of prevention activities and responsible bodies is related to measurement and accountability issues (Hunter, 1990; Ingram *et al.*, 2012; Cinquini *et al.*, 2014; Noto *et al.*, 2023). First, differently from other healthcare services whose outcomes refer to individuals’ health, prevention activities contribute to community-outcomes – i.e. we do not know who will benefit directly from the services. Second, the outcomes of health prevention may refer to the non-happening of an undesirable event (e.g. an epidemic, pandemic, etc.). Third, the long latency of some diseases and the long period that may exist between prevention interventions and impact determine significant delays in the achievement and measurement of outcomes. Fourth, multiple factors outside the direct control of the healthcare system (e.g. epidemiological characteristics, lifestyles, etc.) make it challenging to determine and isolate the contribution of the activity performed by organisations within the health and social care systems. Fifth, prevention results depend on the activity of multiple stakeholders, both public and private ones. Due to these factors, existing PM frameworks for prevention activities are mainly focused on the output indicators (e.g. number of inspections, number of vaccines delivered, etc.) accountable to every actor involved (Schwartz and Deber, 2016), neglecting to consider their impact with respect to the achievement of population outcomes. This narrow approach limits the ability of decision-makers to evaluate the long-term effectiveness of prevention policies and strategies.

To bridge this gap, this research proposes the adoption of simulation techniques. Simulation may support decision-makers in understanding how addressing intermediate results (i.e. output), may contribute to the long-term end-results (outcome) achievement. As such, simulation allows testing alternative policy options and scenarios when real experimentation would be impossible due to the characteristics of the analysed system (Sterman, 2014) – long latency of diseases, delay between actions and impacts, institutional fragmentation, etc.

To address the research objective, this study adopts System Dynamics (SD) – a simulation technique developed by J.W. Forrester (1958) to cope with industrial and social complex systems and widely applied to population health issues (Homer and Hirsch, 2006; Holmström *et al.*, 2022; Noto, 2023). By adopting System Dynamics (SD), the study aims to model the complex interdependencies within the prevention setting and explore how intermediate outputs contribute to long-term health outcomes. A SD model is developed to explore an illustrative case concerning the management of chronic care conditions in a Beveridge healthcare system. Through the discussion of the obtained findings, the research aims to contribute to the refinement of PM practices in prevention services, fostering more effective decision-making and resource allocation in public health.

The article is structured as follows. The next section investigates the literature on PM and public health prevention and the adoption of simulation approach in PM. Building on these foundations, the study introduces an SD approach as a methodological framework for enhancing PM and governance in prevention activities. This approach is then tested through an application to a hypothetical case study, focusing on the management of a typical chronic disease pathway within the Italian healthcare system. In the last section of the article, the results allow to discuss the main advantages of the proposed approach also in comparison with the gaps found in the literature. Finally, the article outlines key theoretical and practical implications and concludes by suggesting directions for future research.

## Theoretical background

The introduction of PM in the public sector has improved the efficiency, effectiveness and transparency of government actions. Introduced after the wave of New Public Management reforms that brought private sector management practices into the public sector, PM is a key approach to monitoring and evaluating the performance of public organisations, aiming at ensuring optimal use of limited resources and improving the quality of services provided (Hood, 1991).

However, the public sector presents unique challenges compared to the private sector (Buttigieg *et al.*, 2016), including the complexity of organisational structures, the multiplicity of stakeholders involved and the often controversial nature of the services provided (Landrum and Baker, 2004). In addition, many problems faced by the public sector are referred to as “wicked problems” (Rittel and Webber, 1973; Weber and Khademian, 2008), as they are characterised by inherent complexity, a lack of clarity about causes and solutions, and the involvement of multiple actors with conflicting interests (Head and Alford, 2015). As pointed out by Noto and colleagues (2023), the term “wicked” does not mean “evil” but rather highlights the challenge of reaching a shared understanding and definition of societal problems. In particular, the “wickedness” of these issues primarily stems from three factors: social pluralism, referring to the coexistence of diverse stakeholder interests and values; institutional complexity, which encompasses the horizontal fragmentation and multilevel governance of public administrations; and scientific uncertainty, arising from gaps in reliable knowledge (Head and Alford, 2015).

In contexts characterised by “wickedness”, measuring and managing performance is particularly challenging as the objectives and targets to be achieved are ill-defined since there is not a shared understanding of the problems to be tackled (Head and Alford, 2015; Noto *et al.*, 2023).

Even in the health sector, and particularly in the context of prevention, these “wicked problems” are particularly prevalent (Blackman *et al.*, 2006). Prevention activities can be classified into two main categories, i.e. primary prevention and secondary prevention (Thompson *et al.*, 1995; Kabir *et al.*, 2007). Primary prevention aims to prevent the onset of chronic conditions, such as diabetes, by addressing risk factors and promoting healthy behaviours before the disease develops (Schulze and Hu, 2005; Bauer *et al.*, 2014). On the other hand, secondary prevention focuses on managing an already diagnosed condition, with the goal of preventing further complications and progression (WHO, 2006; Pedretti *et al.*, 2023). This involves regular monitoring, medication adherence, lifestyle modifications, and other strategies to maintain health and prevent the worsening of the condition (WHO, 2003; Pedretti *et al.*, 2023). Managing these activities becomes difficult. In fact, planning and implementing prevention activities often requires trade-offs between investments in public health and expenditure in current outpatient and inpatient services as well as trade-offs between the health systems and other public sectors’ priorities. Decision makers that aim to control health expenditure may be resistant to allocate resources to prevention and public health activities as the return on investment is long-term and longer than legislative terms, thereby less favoured by policymakers. Results of prevention also depend on other factors such as changes in lifestyle and individual behaviours, epidemiology factors, and so on. Even though prevention activities produce efficiency and saving in the long run, their effects are difficult to be measured accurately (Cinquini *et al.*, 2014; Ferri *et al.*, 2022), as their effects are not immediately observable. It is difficult to determine how many people will actually avoid the occurrence of a disease or an acute episode as a result of a specific preventive intervention – and, thus, what the differential outcome may be. Therefore, traditional PM systems, which are based on static indicators and/or short-term results (Bianchi, 2010; Schwartz and Deber, 2016), are not adequate to comprehensively monitor prevention activities nor to accurately assess their future impacts on the population and the healthcare system.

All these problems are characterised by high complexity, uncertainty, divergence of interests, but also high interdependency and for these reasons considered “wicked problems”. High complexity in that, as the Covid-19 pandemic taught us, every health issue can have social, environmental and economic impacts (Mofijur *et al.*, 2021); uncertainty linked to the dynamic and changing nature of diseases, but also to people’s behaviour; furthermore, the interests of the stakeholders involved (such as patients, health professionals, governments, pharmaceutical organisations, and so on) may be conflicting (Kaplan and Babad, 2011); eventually, the implementation of prevention policies could influence the distribution of resources for the treatment and management of other diseases (Beaglehole *et al.*, 2008).

To synthesise the literature on PM in healthcare and identify the gap this article aims to address, Table 1 presents a classification of previous studies.

**Table 1:** Literature review on performance management in healthcare

Studies	Setting	PM focus	Indicators	Level
Aidemark (2001), Lega and Vendramini (2008), Nuti *et al.* (2018), and others	Inpatient and outpatient care	Output and outcome	Actionable	Organization
Landrum and Baker (2004), Ingram *et al.* (2012), Schwartz and Deber (2016), and others	Public health	Outcome	Non-actionable	Health system
Cinquini *et al.* (2014), Ferri *et al.* (2022), and others	Prevention	Output	Actionable	Organization

**Source(s):** Authors’ own elaboration

As already noted, most of the literature on performance management focuses on inpatient and outpatient care settings, developing actionable indicators to enhance organisational performance in terms of outputs and outcomes achieved (see for instance Nuti *et al*., 2018). Regarding studies related to the public health setting, the majority concentrate on outcome indicators at the health system level (Schwartz and Deber, 2016). However, due to the complexity arising from the “wickedness” of the context, these indicators are not used to assign responsibility at the organisational level, but rather to monitor public health results at the system level. Nonetheless, a specific group of studies focuses on performance management in the prevention setting, developing actionable indicators centred on the activities implemented and their outputs (Cinquini *et al*., 2014; Ferri *et al*., 2022). A gap in the literature thus exists in advancing solutions to address performance management in the prevention setting, particularly in relation to managing the uncertainty surrounding the achievement of health outcomes through specific activities and decisions.

To cope with complexity and uncertainty that characterise the “wicked problems”, several scholars have suggested the adoption of system thinking and simulation techniques (Sterman, 2000, 2014; Datta and Christopher, 2011; Bianchi *et al*., 2017). Simulation models in management studies and practice have an established history starting from the early 1950s (Forrester, 1958). Simulation models can provide practical support for exploring and testing the performance of complex social systems and thus understanding their functioning (Sterman, 2000). In addition, simulation allows alternative scenarios to be tested and the sensitivity of a system to specific variables or parameters to be tested. This approach is of great help when scientific knowledge of valuable variables and parameters is unclear or incomplete (e.g. the contagiousness of a variant of a virus). According to Sterman (2002), “*simulation is essential for effective systems thinking, even when we are faced with an unstructured problem*”.

Simulation techniques have found great application in the field of so-called operations management, which is that branch of business studies that deals with the development, monitoring and implementation of business processes. Traditionally, simulation in operations management is usually associated with the study and analysis of discrete (i.e. not proceeding in the continuum) events, typically characterised by queues and probabilistic distributions for parameters such as arrival and service times (Fowler, 1999).

Since the end of the last century, operational management scholars have also felt the need to embrace simulation techniques that would allow them to consider aspects related to strategy and strategic management (Noto and Cosenz, 2021). Thus, techniques such as System Dynamics (SD) and Agent-Based Modelling (ABM) have become increasingly popular in management and policy studies (Morecroft, 1984; Warren, 1999; Wall, 2016). Both methods apply computer simulation techniques to study complex social systems.

In the field of PM, several studies have focused on the use of SD to support the analysis of organisational performance and the design of systems for managing and measuring it (Cosenz and Noto, 2016). The reasons for scholars’ focus on this specific technique lie in the fact that unlike other approaches, SD adopts a systemic perspective that implies the inclusion of all relevant elements that contribute to the implementation of the strategy and its consequences. Moreover, simulation enable learning through feed-forward mechanisms (Fowler, 1999) that allow decision makers to anticipate expected results of strategies and actions before their implementation.

Based on these premises, this article aims at investigating the support of simulation in fostering PM in “wicked” context such as the health prevention one.

## Method

SD is a methodological approach developed for modelling and simulating complex physical and social systems and experimenting with the models to design strategies and policies (Forrester, 1958). By modelling the interactions between different variables, such as environmental, behavioural and socio-economic factors, this systemic approach is instrumental in understanding how decisions impact at the system level. This holistic perspective focuses on incorporating cause-and-effect circuits, time lags and nonlinear interactions to understand the functioning of the business and inter-organisational system (Meadows, 1980; Sterman, 2000; Bianchi, 2016). SD, in fact, is not an approach geared toward exact prediction of future outcomes, but a technique aimed at understanding the functioning of a social system by linking its structural characteristics with its performance (Meadows, 1980). It provides a systemic perspective and a set of conceptual tools that enable one to frame the structure and behaviour of complex, non-linear, multi-loop feedback systems (Forrester, 1958; Meadows, 1980; Sterman, 2000).

**Figure 1:**
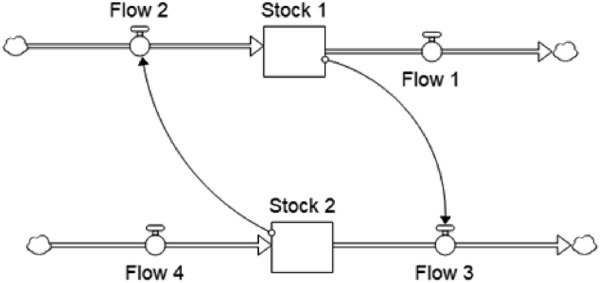
An example of a feedback loop. Source: Authors’ own elaboration

In particular, SD is a valuable tool for analysing the dynamic tendencies of complex systems – i.e. what kind of behavioural patterns they may generate over time. The main assumption of the SD paradigm is that these patterns arise from the causal structure of the system under observation, seen as a closed boundary, i.e. embodying all main relevant variables related to the phenomenon being investigated. This perspective allows the analyst to build closed chains of causal relationships, known as “feedback loops” (Figure 1). SD models are thus made up of several feedback loops linked to each other that contribute to explaining and describing how and why the organisational system behaves according to certain reported trends.

To represent complex and dynamic systems, SD models adopt a graphical syntax in which flow (rate) and stock (level) variables can be distinguished and combined into stock-and-flow diagrams (Größler *et al.*, 2008). By quantifying variables and causal linkages between variables, a system of differential equations is created that can be simulated by numerical algorithms (Sterman, 2000).

Once the simulation model has been developed, calibrated, and tested whether it realistically behaves, inputs are modified to conduct “what if” analyses of how short- and long-term results would change in response to alternative strategy scenarios (Kunc and O’Brien, 2017; Torres *et al.*, 2017).

By virtue of its model building processes allowing scholars and professionals to deal with feedback loops, accumulation and depletion processes, and delays that commonly characterise value creation and delivery in social system, SD has been widely and successfully applied to the healthcare sector (Davahli *et al.*, 2020; Holmström *et al.*, 2022; Cosenz *et al.*, 2024) and, in particular, to public health (Homer *et al.*, 2004; Homer and Hirsch, 2006; Pedamallu *et al.*, 2012; Noto *et al.*, 2023). The reasons for this successful combination are mainly related to the systemic approach, to the possibility of including epidemiology characteristics and non-linear effects of the related variables in SD models, and to the possibility of testing in advance policy effects through simulation (Homer and Hirsch, 2006; Noto, 2023).

SD-based models can help to understand the interactions between different factors influencing health prevention, including individual behaviour, public policies and access to health services (Homer and Hirsch, 2006). These models make it possible to explore alternative scenarios and assess the impacts of health policy decisions over time, enabling decision-makers to make more informed and effective decisions (Homer and Hirsch, 2006; Holmström *et al.*, 2022).

In order to explain how simulation can provide support to deal with “wicked problems” such as the management of prevention activities, a hypothetical case of the management of a typical chronic disease pathway in an Italian context is developed. In particular, the authors adopted the modelling process suggested by Wolstenholme (1990) that identify three stages, namely: construction diagram analysis, phase simulation (stage 1) and phase simulation (stage 2). Being an illustrative case, the validation of the causal structure of the model (see Barlas, 1996) was carried out through direct structure test – in particular structure confirmation test was carried out – and through structure-oriented behaviour tests – in particular extreme condition tests were developed. The SD software used to develop the study is Stella Architect®

## Results

The Italian National Health System is structured on a regional basis and guarantees universal access to comprehensive and essential health services to all citizens (France *et al.*, 2005). It is mainly financed by national and regional taxes through a Beveridge-like system. The government distributes resources to each region according to their population, adjusted on the basis of certain criteria (such as age). Health services are delivered regionally through: (1) Local Health Authorities (LHAs), territorial organisations financed on a capitated basis, which provide primary and public health care directly, as well as secondary and specialised care through their own organisations or by purchasing services from public hospitals or accredited private providers; (2) public and private accredited hospitals, specialised in delivering outpatient and inpatient services (France *et al.*, 2005).

At the local level, LHAs play a central role in implementing national and regional prevention strategies. Each LHA manages a Prevention Department, divided into operational units specialised in different areas, such as workplace safety, food hygiene, veterinary health and prevention of infectious diseases. In addition, LHAs are responsible for primary care services by contracting General Practitioners (GPs).

The SD model was constructed by making assumptions regarding the incidence of a disease within a population, the natural progression of the disease at different stages of development, and the effect of primary care’s caring for framed patients on the course of the disease (*see Appendix.docx (v1.0)*).

**Figure 2:**
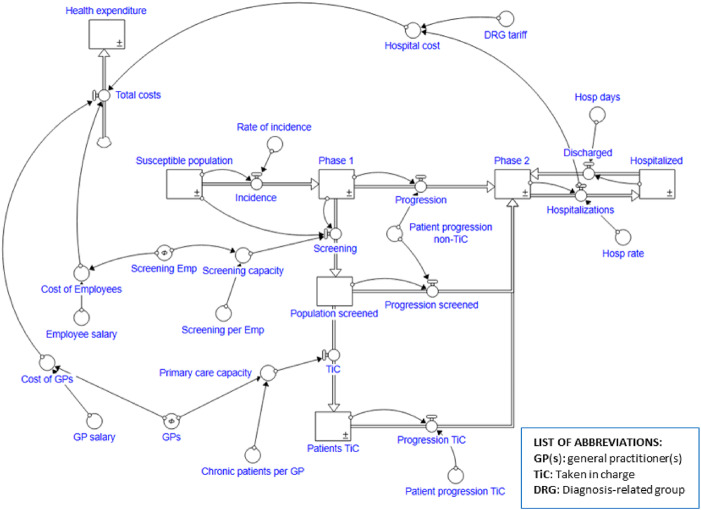
The chronic care pathway – an SD model. Source: Authors’ own elaboration

The Stock-Flow diagram structure of the model is shown in Figure 2.

As shown in Figure 2, the chronic patient pathway through its different phases is represented by the sequence of stock variables shown horizontally – i.e. Reference Population, Phase 1, Phase 2 and Inpatient. The last phase, i.e. hospitalisation, represents the acute manifestation of the pathology, which, from the point of view of prevention, we can interpret as the negative outcome of the system, both from the point of view of the health condition – the acute episode can also lead to serious health outcomes – and from the economic point of view – hospitalisation consumes a large amount of resources represented by the Diagnosis-related group (DRG) cost.

In order to intervene proactively and slow down the natural course of the pathology – and thus decrease the rate of hospitalisation – the health system, and in particular the local authority, has the task of identifying chronic sufferers through a screening campaign. If carried out efficiently and effectively, the latter should allow the primary care setting, i.e. general practitioners (GPs), to take care of patients in a timely manner through the development of an individual care plan and the monitoring of adherence to it. Proper care has the effect of slowing down the natural course of the disease and thus reducing the manifestation of acute episodes and hospitalisations.

Going on to identify the hospitalisation rate (i.e. the ratio of in-patients to the total population) as an outcome indicator, it is interesting to understand how a different allocation of resources, and thus costs, can influence health outcomes. The three main cost categories reported in the model are: the costs of the personnel in charge of screening (prevention setting), the costs of general practitioners (primary care setting) and the costs of admissions calculated through DRG costs (hospital setting).

### Simulation phase (stage 1)

In order to answer the question raised, i.e. what is the best allocation of resources for the improvement of outcomes, a simulation interface was developed in which it is possible to test the effects of a greater/lower allocation of resources, in terms of personnel, between the prevention and primary care settings – i.e. the hospital setting was not considered as the expenditure of this setting is an effect and not a cause of the health outcome.

**Figure 3:**
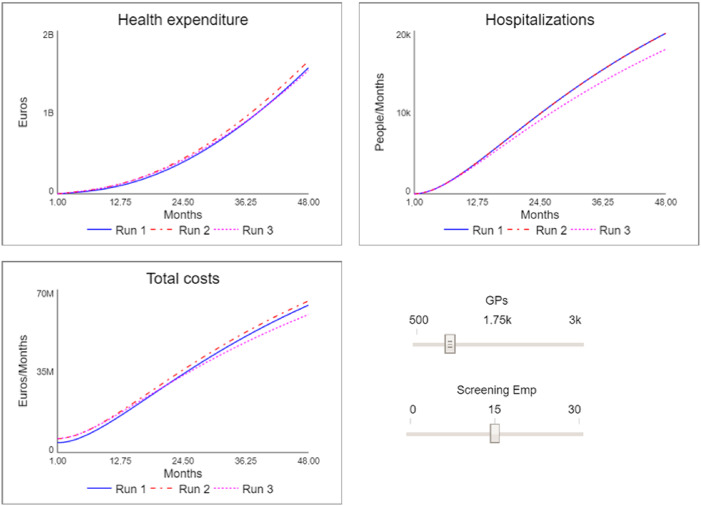
The chronic care simulator. Source: Authors’ own elaboration

In Figure 3, the interface of the simulator is illustrated with the results for four distinct scenarios, namely:

1. Run 1: “as-is” scenario, i.e. the initial conditions of the model in which the number of staff for both screening and primary care activities is understaffed.
2. Run 2: “primary care” scenario, in which the number of GPs is increased from the initial 700 to 1,000.
3. Run 3: “screening” scenario, in which, while maintaining the number of GPs at 1,000, the number of staff units dedicated to screening is increased from 5 to 15.

As can be observed, the Run 2 scenario, in which the primary care setting is strengthened by increasing the number of GPs, despite the fact that it entails higher costs, and an overall higher expenditure, has a negative impact on hospitalisations and therefore on the outcome results. Going to examine the model and the partial results, it is possible to understand how this result derives from the fact that, although the capacity for taking on chronic patients has increased, only a part of the latter has been identified because the capacity of the screening activity is under-dimensioned.

This explanation is even more evident when looking at the results of the Run 3 scenario. Although the initial costs of this scenario are still higher (due to the higher number of staff employed), it is observed that at the end of the period considered, they are lower than in the previous scenarios; this is because the cost of admissions has been reduced due to a reduction in hospitalisations. More important is the significant improvement in terms of performance. The outcome result expressed in terms of reduced hospitalisations is in fact lower (and therefore better) than in the two previous scenarios. This means that fewer people experienced acute episodes, with the risks that these entail.

**Figure 4:**
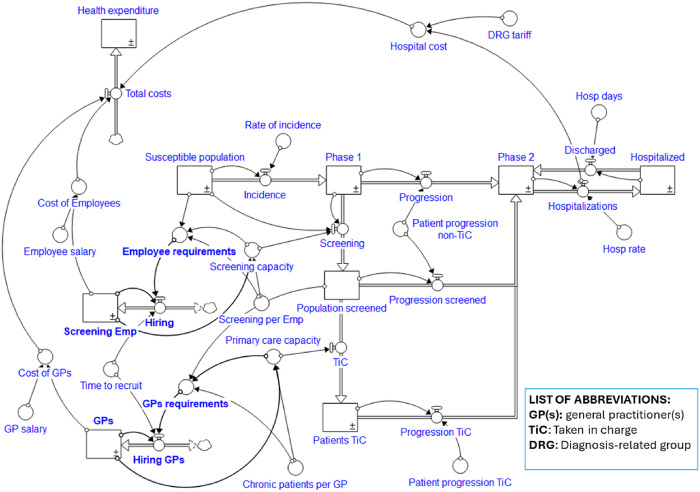
The chronic care pathways redesigned based on the “endogenisation” process. Source: Authors’ own elaboration

Based on the findings from the first phase of the simulation, it is possible to review the causal structure of the model, considering the system and its key variables as endogenous. The allocation of resources across various settings must be determined based on the needs of the healthcare system. Therefore, the staffing of screening and primary care activities should depend on information from the system itself – i.e. service demand and capacity. This “endogenisation” process is illustrated graphically in Figure 4, where changes to the previously reported model are highlighted in bold.

As can be seen from Figure 4, the decision-making processes with regard to the recruitment of personnel for screening and GP activity is now determined on the basis of an actual need that depends on the demand for the respective services and by the existent operational capacity.

### Simulation phase (stage 2)

**Figure 5:**
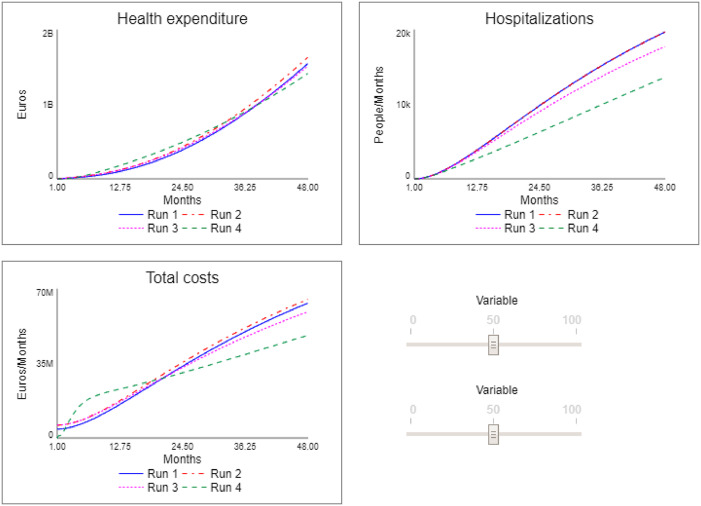
The chronic care simulator – Run 4. Source: Authors’ own elaboration

To test the results of the new causal structure, a second phase of simulation was carried out. Figure 5 depicts the results of Run 4, which represents the simulation of the chronic care model after the “endogenisation” process.

As can be observed from Figure 5, the results obtained through this “endogenisation” process led to improved results. Regarding expenditure, while the total cost increases significantly in the short term due to the recruitment of new staff, in the medium term – i.e. during the second year – costs decrease below the level of the previous simulations. Overall, this results in total expenditure over the four years considered being lower than in the scenarios of Run 1, 2, and 3. On the other hand, health outcomes also improve significantly, as the hospitalisation rate is far below that of the previously simulated scenarios.

This “endogenisation” can be drived through the development and design of “system” performance indicators (PI) leading toward a better allocation of resources. In this specific case, it is possible to define three performance indicators that inform the decision-makers with regard to staffing needs in the various settings. Table 2 reports the indicators that are directly derived from the reviewed model.

**Table 2:** Key performance indicators

Indicator	Numerator	Denominator	Target
Outcome PI	Hospitalizations	Total population	Run 4
Screening PI	Screening capacity	Reference population	1
Primary care PI	GPs capacity	Screened population	1

**Source(s):** Authors’ own elaboration

As one may notice from Table 2, the outcome PI, that measures the hospitalization rate, is the key outcome indicator that should be monitored over time to check whether the results obtained are consistent with the simulated scenarios (in particular with Run 4 results).

With regard to screening, one indicator can be determined by relating screening capacity – measured as the numbers of employees dedicated to screening multiplied by their average productivity – to the reference population. Concerning primary care, the indicator can be constructed by relating the productive capacity of the GPs – measured as the numbers of GPs times their average ability to take in charge chronic patients effectively – to the stock relative to the selected population. Both indicators should aim for a value of 1 to ensure optimal outcome performance.

## Discussion

The analysis conducted in the Italian prevention system highlights the importance of a balanced allocation of resources among different healthcare settings to optimize health outcomes and cost-effectiveness. The Italian National Health System, structured on a regional basis, ensures universal access to essential health services, with resources allocated based on demographic criteria. LHAs play a crucial role in implementing prevention strategies, including disease screening and primary care management through General Practitioners. The SD model developed in this study illustrates the chronic patient pathway and evaluates how different resource allocations impact hospitalisation rates, a key outcome indicator.

The simulation results across three scenarios emphasize the significance of an integrated approach. The “as-is” scenario reflects an understaffed healthcare system with suboptimal performance. In the “primary care” scenario, increasing the number of GPs led to a higher overall expenditure but failed to significantly reduce hospitalisations due to insufficient screening capacity. Conversely, in the “screening” scenario, enhancing screening efforts resulted in lower hospitalisation rates and a reduction in long-term healthcare costs. This outcome confirms that early identification and timely management of chronic patients are crucial for mitigating acute episodes and preventing hospital admissions. The findings suggest that a more effective allocation of resources between screening and primary care settings can enhance system performance and reduce costs in the long term. The redesign of the chronic care pathway based on an “endogenisation” process highlights the necessity of adjusting staffing levels dynamically in response to system demands, fostering a more data-driven decision-making approach. The simulation related to the “endogenisation” scenario confirms that an informed decision-making process, facilitated by a set of tailored performance indicators, can lead to improved results in terms of both outcomes and expenditure.

The study underscores the utility of simulation models in predicting the impact of strategic healthcare decisions concerning resource allocation, particularly in those contexts characterised by wicked problems such as prevention. From the analysis of the findings is possible to draw interesting conclusions.

Firstly, the simulation makes it possible to assess in advance the effects in terms of performance of strategic decisions (in the specific case concerning the allocation of resources) by activating the feed-forward perspective previously mentioned (Fowler, 1999).

Secondly, an interesting insight offered by the example relates to the need to consider the system, and its variables, as endogenous. The allocation of resources between the various settings must be determined on the basis of the needs of the healthcare system; therefore, the staffing of screening and primary care activities must depend on information from the system itself. This “endogenisation” process is illustrated graphically in Figure 4, where changes to the previously reported model are highlighted in bold.

Finally, the case highlights how at the health system level, considering all settings, a negative relationship between costs and performance may emerge. That is, in certain settings, a reduction in expenditure is determined by an improvement in outcome results. The literature on this specific topic reports conflicting results (Schreyögg and Stargardt, 2010; Nuckols *et al.*, 2013; Spano *et al.*, 2022). According to some studies higher costs are associated with better performance, according to others better performance is associated with lower costs. This discordance depends on many contextual factors of these studies, which mainly refer to the type of service analysed, the cost and performance criteria and the object of analysis (operational unit, health organisations, health system, etc.). In the case of prevention, also on the basis of the illustrative case described above, the underlying relationship of the “prevention lever” (Noto, 2024) would appear to be true, i.e. a correct allocation of resources in prevention determines an improvement in performance which, in the long term, has the effect of reducing overall expenditure.

## Conclusions

This research shows that the adoption of simulation techniques to manage performance in prevention settings may empower decision-makers when dealing with uncertainty related to measurement issues. In particular, by understanding the long-term impacts of prevention initiatives, decision-makers at various levels may redefine PM output-targets to guide organisations toward the achievement of desired and sustainable community-outcomes.

In this way, it becomes possible to deal with prevention not as an isolated phenomenon, but as part of a complex system, where each variable influences the others. The ability to simulate scenarios makes it possible to predict how prevention policies will influence the health of the population and the costs of the health system beyond the short term and therefore in the medium and long-term. Through these models, it is possible to explore different settings, assessing what might happen if, as in our example, the number of staff assigned to primary care or prevention activities is increased. This type of predictive analysis provides a solid basis for making informed decisions, while knowing that the benefits of prevention may not become apparent until many years later. Furthermore, simulation results can provide a useful benchmark for monitoring performance over time and assessing its systemic impacts. They are not limited to considering the immediate effects, but allow for the analysis of how these policies affect not only health care costs, but also other aspects of the system, such as the reduction of hospitalisations, the prevention of chronic diseases, and the improvement of citizens’ quality of life. This holistic view is crucial in tackling “wicked problems” such as public health, where each intervention fits into a broader framework of interdependent factors.

In this context, future research may be aimed at developing a real-life case study of a prevention activity, so as to overcome the main limitation of this research is related to the adoption of an illustrative study.

## Appendix

**Table A1:** Model details

Variable	Equation	Properties	Units
*Model 1 variables and formula*			
Costs(t)	Costs(t−dt) + (Total_costs) * dt	INIT Costs = 0	Euros
Hospitalized(t)	Hospitalized(t−dt) + (Hospitalizations−Discharged) * dt	INIT Hospitalized = 0	People
Patients_TiC(t)	Patients_TiC(t−dt) + (TiC−Progression_TiC) * dt	INIT Patients_TiC = 0	People
Phase_1(t)	Phase_1(t−dt) + (Incidence−Progression−Screening) * dt	INIT Phase_1 = 1,000	People
Phase_2(t)	Phase_2(t−dt) + (Progression + Discharged + Progression_TiC + Progression_screened−Hospitalizations) * dt	INIT Phase_2 = 100	People
Population_screened(t)	Population_screened(t−dt) + (Screening−TiC−Progression_screened) * dt	INIT Population_screened = 0	People
Susceptible_population(t)	Susceptible_population(t−dt) + (−Incidence) * dt	INIT Susceptible_population = 1,000,000	People
Discharged	Hospitalized/Hosp_days		People/Months
Hospitalizations	Phase_2*Hosp_rate		People/Months
Incidence	Susceptible_population*Rate_of_incidence		People/Months
Progression	Phase_1*“Patient_progression_non-TiC”		People/Months
Progression_screened	Patient_progression_non-TiC*Population_screened		People/Months
Progression_TiC	Patient_progression_TiC*Patients_TiC		People/Months
Screening	Screening_capacity*Phase_1/Susceptible_population		People/Months
TiC	Primary_care_capacity		People/Months
Total_costs	Hospital_cost + Cost_of_Employees + Cost_of_GPs		Euros/Months
Chronic_patients_per_GP	5		People/Employees/Year
Cost_of_Employees	Employees*Employee_salary		Euros/Years
Cost_of_GPs	GPs*GP_salary		Euros/Years
DRG_tariff	3,000		Euros/People
Employee_salary	4,000		Euros/Employees/Years
Employees	15		People
GP_salary	6,000		Euros/Employee/Years
GPs	1,000		Employees
Hosp_days	5		Days
Hosp_rate	0.05		Per Year
Hospital_cost	Hospitalizations*DRG_tariff		Euros/Months
Patient_progression_non-TiC	0.1		Per Year
Patient_progression_TiC	0.02		Per Year
Primary_care_capacity	GPs*Chronic_patients_per_GP		People/Years
Rate_of_incidence	0.02		Per Year
Screening_capacity	Screening_per_Emp*Employees		People/Years
Screening_per_Emp	2,000		People/Employees/Years
*Model 2 variables and formula*			
GPs(t)	GPs(t−dt) + (Hiring_GPs) * dt	INIT GPs = 0	Employees
Health_expenditure(t)	Health_expenditure(t−dt) + (Total_costs) * dt	INIT Health_expenditure = 0	Euros
Hospitalized(t)	Hospitalized(t−dt) + (Hospitalizations−Discharged) * dt	INIT Hospitalized = 0	People
Patients_TiC(t)	Patients_TiC(t−dt) + (TiC−Progression_TiC) * dt	INIT Patients_TiC = 0	People
Phase_1(t)	Phase_1(t−dt) + (Incidence−Progression−Screening) * dt	INIT Phase_1 = 1,000	People
Phase_2(t)	Phase_2(t−dt) + (Progression + Discharged + Progression_TiC + Progression_screened−Hospitalizations) * dt	INIT Phase_2 = 100	People
Population_screened(t)	Population_screened(t−dt) + (Screening−TiC−Progression_screened) * dt	INIT Population_screened = 0	People
Screening_Emp(t)	Screening_Emp(t−dt) + (Hiring) * dt	INIT Screening_Emp = 0	Employees
Susceptible_population(t)	Susceptible_population(t−dt) + (−Incidence) * dt	INIT Susceptible_population = 1,000,000	People
Discharged	Hospitalized/Hosp_days		People/Months
Hiring	(Employee_requirements-Screening_Emp)/Time_to_recruit		Employees/Months
Hiring_GPs	(GPs_requirements-GPs)/Time_to_recruit		Employees/Months
Hospitalizations	Phase_2*Hosp_rate		People/Months
Incidence	Susceptible_population*Rate_of_incidence		People/Months
Progression	Phase_1*“Patient_progression_non-TiC”		People/Months
Progression_screened	Patient_progression_non-TiC*Population_screened	OUTFLOW PRIORITY: 2	People/Months
Progression_TiC	Patient_progression_TiC*Patients_TiC		People/Months
Screening	Screening_capacity*Phase_1/Susceptible_population		People/Months
TiC	Primary_care_capacity	OUTFLOW PRIORITY: 1	People/Months
Total_costs	Hospital_cost + Cost_of_Employees + Cost_of_GPs		Euros/Months
Chronic_patients_per_GP	5		People/Employees/Year
Cost_of_Employees	Screening_Emp*Employee_salary		Euros/Years
Cost_of_GPs	GPs*GP_salary		Euros/Years
DRG_tariff	3,000		Euros/People
Employee_requirements	(Susceptible_population-Screening_capacity)/Screening_per_Emp		Employees
Employee_salary	4,000		Euros/Employees/Years
GP_salary	6,000		Euros/Employee/Years
GPs_requirements	(Population_screened-Primary_care_capacity)/Chronic_patients_per_GP		Employees
Hosp_days	5		Days
Hosp_rate	0.05		Per Year
Hospital_cost	Hospitalizations*DRG_tariff		Euros/Months
Patient_progression_non-TiC	0.1		Per Year
Patient_progression_TiC	0.02		Per Year
Primary_care_capacity	GPs*Chronic_patients_per_GP		People/Years
Rate_of_incidence	0.02		Per Year
Screening_capacity	Screening_per_Emp*Screening_Emp		People/Years
Screening_per_Emp	2,000		People/Employees/Years
Time_to_recruit	0.5		Years

### Extreme condition test

Run 1 – As is
Run 2 – No employees nor GPs
Run 3 – Total population = 0
